# 1-[(4-*tert*-Butyl­phen­yl)sulfon­yl]-1*H*-benzimidazole

**DOI:** 10.1107/S1600536811005551

**Published:** 2011-02-26

**Authors:** K. B. Abdireymov, N. S. Mukhamedov, R. Ya. Okmanov, M. J. Ayimbetov, Kh. M. Shakhidoyatov

**Affiliations:** a S. Yunusov Institute of the Chemistry of Plant Substances, Academy of Sciences of Uzbekistan, Mirzo Ulugbek Str. 77, Tashkent 100170, Uzbekistan; b Kara-Kalpak State University, Acad. Abdirov Str. 1, Nukus 742000, Uzbekistan

## Abstract

The title compound, C_17_H_18_N_2_O_2_S, was synthesized by aryl­sulfonyl­ation of 1-hy­droxy­methyl-1*H*-benzimidazole in the presence of triethyl­amine. The benzimidazole and benzene rings form a dihedral angle of 84.1 (1)°. The *tert*-butyl group was treated as rotationally disordered over two orientations in a 0.51 (2):0.49 (2) ratio. In the crystal, weak inter­molecular C—H⋯O hydrogen bonds link the mol­ecules into chains propagating in [010].

## Related literature

For the biological and pharmaceutical properties of benzimidazole derivatives, see: Koči *et al.* (2002 ▶); Matsuno *et al.* (2000 ▶); Garuti *et al.* (1999 ▶). For related structures, see: Rashid *et al.* (2006 ▶, 2007 ▶). For the aryl­sulfonyl­ation of benzimidazole derivatives, see: Abdireimov *et al.* (2010 ▶).
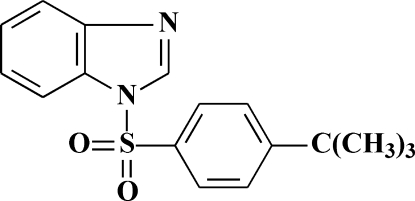


## Experimental

### 

#### Crystal data

C_17_H_18_N_2_O_2_S
*M* *_r_* = 314.39
Monoclinic, 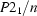

*a* = 12.142 (2) Å
*b* = 8.8940 (18) Å
*c* = 15.324 (3) Å
β = 96.78 (3)°
*V* = 1643.3 (6) Å^3^
*Z* = 4
Cu *K*α radiation
μ = 1.82 mm^−1^
*T* = 302 K
0.60 × 0.25 × 0.22 mm

#### Data collection

Stoe Stadi-4 four-circle diffractometer
Absorption correction: ψ scan (North *et al.*, 1968 ▶) *T*_min_ = 0.609, *T*_max_ = 0.670
2727 measured reflections
2429 independent reflections
1813 reflections with *I* > 2σ(*I*)
*R*_int_ = 0.044
θ_max_ = 60.0°
3 standard reflections every 60 min  intensity decay: 5.2%

#### Refinement

*R*[*F* ^2^ > 2σ(*F* ^2^)] = 0.058
*wR*(*F* ^2^) = 0.150
*S* = 1.12
2429 reflections
234 parameters
H-atom parameters constrained
Δρ_max_ = 0.15 e Å^−3^
Δρ_min_ = −0.27 e Å^−3^

### 

Data collection: *STADI4* (Stoe & Cie, 1997 ▶); cell refinement: *STADI4*; data reduction: *X-RED* (Stoe & Cie, 1997 ▶); program(s) used to solve structure: *SHELXS97* (Sheldrick, 2008 ▶); program(s) used to refine structure: *SHELXL97* (Sheldrick, 2008 ▶); molecular graphics: *XP* (Bruker, 1998 ▶); software used to prepare material for publication: *publCIF* (Westrip, 2010 ▶).

## Supplementary Material

Crystal structure: contains datablocks I, global. DOI: 10.1107/S1600536811005551/cv5052sup1.cif

Structure factors: contains datablocks I. DOI: 10.1107/S1600536811005551/cv5052Isup2.hkl

Additional supplementary materials:  crystallographic information; 3D view; checkCIF report

## Figures and Tables

**Table 1:** Hydrogen-bond geometry (Å, °)

*D*—H⋯*A*	*D*—H	H⋯*A*	*D*⋯*A*	*D*—H⋯*A*
C7—H7*A*⋯O2^i^	0.93	2.56	3.430 (5)	156
C13—H13*A*⋯O1^i^	0.93	2.56	3.292 (5)	136

Symmetry code: (i) 
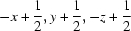
.

**Fig. 1.**
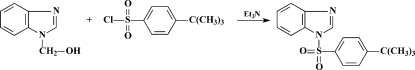
Reaction sequence.

**Fig. 2.**
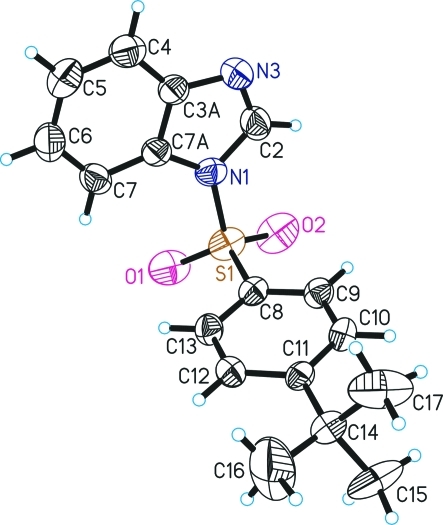
The molecular structure of the title compound, showing the atomic numbering scheme. The displacement ellipsoids are drawn at the 30% probability level. Minor parts of the disordered atoms were omitted for clarity.

## supplementary crystallographic information

### Comment

Benzimidazole derivatives are important heteroaromatic compounds which have attracted great attention due to their biological and pharmaceutical activities (Koči *et al.*, 2002; Matsuno *et al.*, 2000; Garuti *et al.*, 1999). The title compound has been obtained by the reaction of hydroxymethyl-1*H*-benzimidazole with 4-*tert*-butilbenzolsulfochlorid  in the presence of triethylamine (Abdireimov *et al.*, 2010; see also Fig. 1), where occurs deformylation of oxymethyl groups. The structure of the final product was investigated by ^1^H NMR-spectroscopy and X-ray diffraction.

The molecule (Fig. 2) consist of two flat fragments - benzimidazolic (N1/C2/N3/C3A–C7A) and benzolic (C8–C13) (r.m.s. deviation = 0.0082 and 0.0017 Å) ones, which form a dihedral angle of 84.1 (1)°. All bond lengths and angles are normal and comparable with those in related structures  (Rashid *et al.*, 2006, 2007). The *tert*-butyl group was rotationally disordered over two orientations with occupancies refined to 0.51 (2) and 0.49 (2), respectively.

In the crystal structure, weak intermolecular C—H···O hydrogen bonds (Table 1) link the molecules into chains propagated in direction [010].

### Experimental

To three-necked flask supplied with a mixer, containing solution of 2.32 g (10 mmol) 4-*tert*-butilbenzolsulfochloride in 15 ml acetone is added mixture of 1.48 g (10 mmol) 1-hydroxymethyl-1*H*benzimidazole and 1.01 g (10 mmol) triethylamine in 30 ml of acetone. The reaction mixture is mixed at room temperature for 4 h and acetone is evaporated. The rest of mass is washed by 100 ml of water and filtered, and recrystallized from benzine and received 2.61 g (76%) of title compound, m.p. 395–396 K.

The colorless crystals suitable for *x*-ray analysis have been grown from absolutized ethanol at room temperature.

^1^H NMR (400 MHz, CDCl_3_): 8.33 (1*H*,s, H-2), 7.85 (3*H*, m, H-4, 5, 6,), 7.71 (1*H*, dd, J═2.1, J═8.2 Hz H-7), 7.45 (2*H*, m, H-9, 13), 7.32 (2*H*, m, H-10, 12), 1.20 (9*H*, s, C(CH_3_)_3_).

### Refinement

The C15, C16 and C17 methyl carbon atoms of a *tert*-butyl group were treated as rotationally disordered over two orientations with site occupancy factors refined to 0.51 (2) and 0.49 (2), respectively. All H atoms were positioned geometrically and treated as riding atoms, with C—H distances of 0.96 Å for CH_3_, 0.93 Å for C_ar_ and included in the refinement in a riding motion approximation, with *U*_iso_=1.2*U*_eq_(C) or *U*_iso_=1.5*U*_eq_(C) for methyl H atoms.

### Crystal data

**Table:**

C_17_H_18_N_2_O_2_S	*F*(000) = 664
*M**_r_* = 314.39	*D*_x_ = 1.271 Mg m^−^^3^
Monoclinic, *P*2_1_/*n*	Melting point: 395(1) K
Hall symbol: -P 2yn	Cu *K*α radiation, λ = 1.54184 Å
*a* = 12.142 (2) Å	Cell parameters from 14 reflections
*b* = 8.8940 (18) Å	θ = 10–20°
*c* = 15.324 (3) Å	µ = 1.82 mm^−^^1^
β = 96.78 (3)°	*T* = 302 K
*V* = 1643.3 (6) Å^3^	Prism, colourless
*Z* = 4	0.60 × 0.25 × 0.22 mm

### Data collection

**Table:**

Stoe Stadi-4 four-circle diffractometer	1813 reflections with *I* > 2σ(*I*)
Radiation source: fine-focus sealed tube	*R*_int_ = 0.044
graphite	θ_max_ = 60.0°, θ_min_ = 4.4°
Scan width (ω) = 1.56 – 1.80, scan ratio 2θ:ω = 1.00 I(Net) and sigma(I) calculated according to Blessing (1987)	*h* = −13→13
Absorption correction: ψ scan (North *et al.*, 1968)	*k* = 0→9
*T*_min_ = 0.609, *T*_max_ = 0.670	*l* = 0→17
2727 measured reflections	3 standard reflections every 60 min
2429 independent reflections	intensity decay: 5.2%

### Refinement

**Table:**

Refinement on *F*^2^	Secondary atom site location: difference Fourier map
Least-squares matrix: full	Hydrogen site location: inferred from neighbouring sites
*R*[*F*^2^ > 2σ(*F*^2^)] = 0.058	H-atom parameters constrained
*wR*(*F*^2^) = 0.150	*w* = 1/[σ^2^(*F*_o_^2^) + (0.0478*P*)^2^ + 1.1618*P*] where *P* = (*F*_o_^2^ + 2*F*_c_^2^)/3
*S* = 1.12	(Δ/σ)_max_ < 0.001
2429 reflections	Δρ_max_ = 0.15 e Å^−^^3^
234 parameters	Δρ_min_ = −0.27 e Å^−^^3^
0 restraints	Extinction correction: *SHELXL97* (Sheldrick, 2008), Fc^*^=kFc[1+0.001xFc^2^λ^3^/sin(2θ)]^-1/4^
Primary atom site location: structure-invariant direct methods	Extinction coefficient: 0.0054 (4)

### Special details

**Table:**

Geometry. All e.s.d.'s (except the e.s.d. in the dihedral angle between two l.s. planes) are estimated using the full covariance matrix. The cell e.s.d.'s are taken into account individually in the estimation of e.s.d.'s in distances, angles and torsion angles; correlations between e.s.d.'s in cell parameters are only used when they are defined by crystal symmetry. An approximate (isotropic) treatment of cell e.s.d.'s is used for estimating e.s.d.'s involving l.s. planes.
Refinement. Refinement of *F*^2^ against ALL reflections. The weighted *R*-factor *wR* and goodness of fit *S* are based on *F*^2^, conventional *R*-factors *R* are based on *F*, with *F* set to zero for negative *F*^2^. The threshold expression of *F*^2^ > σ(*F*^2^) is used only for calculating *R*-factors(gt) *etc*. and is not relevant to the choice of reflections for refinement. *R*-factors based on *F*^2^ are statistically about twice as large as those based on *F*, and *R*- factors based on ALL data will be even larger.

### Fractional atomic coordinates and isotropic or equivalent isotropic displacement parameters (Å^2^)

**Table:**

	*x*	*y*	*z*	*U*_iso_*/*U*_eq_	Occ. (<1)
S1	0.15656 (9)	0.56256 (13)	0.30146 (7)	0.0817 (4)	
O1	0.2287 (3)	0.6147 (4)	0.24143 (18)	0.1059 (11)	
O2	0.0939 (3)	0.4275 (3)	0.2866 (2)	0.1116 (11)	
N1	0.2366 (2)	0.5318 (4)	0.39620 (19)	0.0695 (8)	
N3	0.2705 (3)	0.4528 (4)	0.5352 (3)	0.0882 (11)	
C2	0.2092 (4)	0.4382 (5)	0.4623 (3)	0.0855 (12)	
H2B	0.1509	0.3700	0.4542	0.103*	
C3A	0.3453 (3)	0.5650 (4)	0.5199 (3)	0.0678 (10)	
C4	0.4313 (4)	0.6232 (5)	0.5767 (3)	0.0857 (12)	
H4A	0.4460	0.5875	0.6340	0.103*	
C5	0.4939 (4)	0.7339 (6)	0.5467 (4)	0.0924 (13)	
H5A	0.5514	0.7757	0.5845	0.111*	
C6	0.4741 (4)	0.7862 (5)	0.4611 (4)	0.0907 (13)	
H6A	0.5189	0.8618	0.4425	0.109*	
C7	0.3901 (3)	0.7286 (4)	0.4032 (3)	0.0764 (11)	
H7A	0.3773	0.7625	0.3455	0.092*	
C7A	0.3259 (3)	0.6188 (4)	0.4344 (2)	0.0621 (9)	
C8	0.0676 (3)	0.7078 (4)	0.3232 (2)	0.0593 (9)	
C9	−0.0313 (3)	0.6758 (4)	0.3528 (2)	0.0639 (9)	
H9A	−0.0507	0.5766	0.3627	0.077*	
C10	−0.1020 (3)	0.7904 (4)	0.3678 (2)	0.0635 (9)	
H10A	−0.1696	0.7674	0.3875	0.076*	
C11	−0.0760 (3)	0.9392 (4)	0.3545 (2)	0.0542 (8)	
C12	0.0251 (3)	0.9681 (4)	0.3244 (2)	0.0673 (10)	
H12A	0.0450	1.0670	0.3146	0.081*	
C13	0.0966 (3)	0.8541 (4)	0.3086 (2)	0.0672 (10)	
H13A	0.1640	0.8759	0.2883	0.081*	
C14	−0.1562 (3)	1.0652 (5)	0.3703 (3)	0.0709 (10)	
C15	−0.2610 (16)	1.044 (3)	0.3059 (11)	0.131 (7)	0.51 (2)
H15A	−0.3100	1.1275	0.3104	0.197*	0.51 (2)
H15B	−0.2974	0.9526	0.3196	0.197*	0.51 (2)
H15C	−0.2413	1.0382	0.2471	0.197*	0.51 (2)
C16	−0.113 (2)	1.2215 (18)	0.361 (3)	0.234 (18)	0.51 (2)
H16A	−0.0440	1.2326	0.3985	0.351*	0.51 (2)
H16B	−0.1658	1.2926	0.3788	0.351*	0.51 (2)
H16C	−0.1015	1.2394	0.3015	0.351*	0.51 (2)
C17	−0.1958 (18)	1.047 (2)	0.4597 (8)	0.142 (10)	0.51 (2)
H17A	−0.1330	1.0447	0.5041	0.214*	0.51 (2)
H17B	−0.2367	0.9549	0.4613	0.214*	0.51 (2)
H17C	−0.2428	1.1302	0.4706	0.214*	0.51 (2)
C15A	−0.0948 (12)	1.1855 (16)	0.4249 (10)	0.093 (5)	0.49 (2)
H15D	−0.1469	1.2544	0.4454	0.140*	0.49 (2)
H15E	−0.0470	1.2386	0.3899	0.140*	0.49 (2)
H15F	−0.0511	1.1403	0.4743	0.140*	0.49 (2)
C16A	−0.248 (2)	1.013 (2)	0.415 (3)	0.233 (19)	0.49 (2)
H16D	−0.2963	1.0962	0.4239	0.350*	0.49 (2)
H16E	−0.2204	0.9706	0.4708	0.350*	0.49 (2)
H16F	−0.2894	0.9380	0.3797	0.350*	0.49 (2)
C17A	−0.192 (3)	1.141 (3)	0.2816 (11)	0.150 (10)	0.49 (2)
H17D	−0.2465	1.2174	0.2891	0.226*	0.49 (2)
H17E	−0.2240	1.0676	0.2403	0.226*	0.49 (2)
H17F	−0.1290	1.1865	0.2600	0.226*	0.49 (2)

### Atomic displacement parameters (Å^2^)

**Table:**

	*U*^11^	*U*^22^	*U*^33^	*U*^12^	*U*^13^	*U*^23^
S1	0.0854 (7)	0.0894 (8)	0.0698 (6)	0.0210 (6)	0.0072 (5)	−0.0219 (6)
O1	0.112 (2)	0.145 (3)	0.0660 (17)	0.044 (2)	0.0306 (16)	−0.0054 (18)
O2	0.116 (2)	0.087 (2)	0.126 (3)	0.0087 (19)	−0.012 (2)	−0.0552 (19)
N1	0.0673 (19)	0.069 (2)	0.0716 (19)	0.0118 (16)	0.0044 (16)	0.0041 (16)
N3	0.083 (2)	0.086 (3)	0.096 (3)	0.001 (2)	0.014 (2)	0.026 (2)
C2	0.080 (3)	0.067 (3)	0.112 (4)	0.000 (2)	0.020 (3)	0.016 (3)
C3A	0.061 (2)	0.068 (2)	0.075 (2)	0.0119 (19)	0.0132 (19)	0.008 (2)
C4	0.082 (3)	0.095 (3)	0.079 (3)	0.012 (3)	0.007 (2)	0.006 (2)
C5	0.071 (3)	0.094 (3)	0.111 (4)	0.004 (3)	0.003 (3)	−0.015 (3)
C6	0.073 (3)	0.075 (3)	0.128 (4)	−0.003 (2)	0.023 (3)	0.006 (3)
C7	0.075 (3)	0.070 (3)	0.087 (3)	0.012 (2)	0.024 (2)	0.019 (2)
C7A	0.059 (2)	0.059 (2)	0.070 (2)	0.0141 (18)	0.0141 (17)	0.0043 (18)
C8	0.057 (2)	0.070 (2)	0.0502 (19)	0.0035 (18)	0.0041 (15)	−0.0080 (17)
C9	0.060 (2)	0.058 (2)	0.071 (2)	−0.0046 (18)	0.0000 (18)	−0.0060 (18)
C10	0.050 (2)	0.071 (2)	0.069 (2)	−0.0058 (18)	0.0079 (16)	−0.0093 (19)
C11	0.0482 (18)	0.062 (2)	0.0502 (18)	−0.0012 (16)	−0.0024 (14)	−0.0048 (16)
C12	0.067 (2)	0.059 (2)	0.076 (2)	−0.0038 (19)	0.0100 (19)	0.0078 (19)
C13	0.058 (2)	0.081 (3)	0.065 (2)	−0.002 (2)	0.0149 (17)	0.005 (2)
C14	0.063 (2)	0.074 (3)	0.074 (2)	0.011 (2)	0.0002 (18)	−0.006 (2)
C15	0.100 (10)	0.177 (19)	0.106 (11)	0.076 (11)	−0.037 (8)	−0.032 (11)
C16	0.17 (2)	0.069 (9)	0.49 (6)	0.027 (10)	0.14 (3)	0.05 (2)
C17	0.20 (2)	0.153 (18)	0.071 (8)	0.102 (16)	0.023 (8)	−0.007 (8)
C15A	0.095 (7)	0.057 (8)	0.122 (9)	0.014 (7)	−0.010 (7)	−0.037 (7)
C16A	0.157 (18)	0.104 (11)	0.48 (5)	−0.015 (13)	0.23 (3)	−0.07 (2)
C17A	0.18 (2)	0.153 (18)	0.105 (10)	0.096 (16)	−0.050 (13)	−0.022 (11)

### Geometric parameters (Å, °)

**Table:**

S1—O1	1.421 (3)	C12—C13	1.375 (5)
S1—O2	1.426 (3)	C12—H12A	0.9300
S1—N1	1.671 (3)	C13—H13A	0.9300
S1—C8	1.742 (3)	C14—C16A	1.455 (17)
N1—C2	1.381 (5)	C14—C16	1.498 (17)
N1—C7A	1.402 (5)	C14—C15A	1.501 (12)
N3—C2	1.274 (5)	C14—C17	1.513 (15)
N3—C3A	1.387 (5)	C14—C15	1.527 (15)
C2—H2B	0.9300	C14—C17A	1.535 (18)
C3A—C4	1.379 (5)	C15—H15A	0.9600
C3A—C7A	1.389 (5)	C15—H15B	0.9600
C4—C5	1.357 (6)	C15—H15C	0.9600
C4—H4A	0.9300	C16—H16A	0.9600
C5—C6	1.386 (6)	C16—H16B	0.9600
C5—H5A	0.9300	C16—H16C	0.9600
C6—C7	1.369 (6)	C17—H17A	0.9600
C6—H6A	0.9300	C17—H17B	0.9600
C7—C7A	1.371 (5)	C17—H17C	0.9600
C7—H7A	0.9300	C15A—H15D	0.9600
C8—C9	1.363 (5)	C15A—H15E	0.9600
C8—C13	1.373 (5)	C15A—H15F	0.9600
C9—C10	1.369 (5)	C16A—H16D	0.9600
C9—H9A	0.9300	C16A—H16E	0.9600
C10—C11	1.381 (5)	C16A—H16F	0.9600
C10—H10A	0.9300	C17A—H17D	0.9600
C11—C12	1.385 (5)	C17A—H17E	0.9600
C11—C14	1.523 (5)	C17A—H17F	0.9600
			
O1—S1—O2	122.0 (2)	C13—C12—H12A	119.2
O1—S1—N1	106.03 (18)	C11—C12—H12A	119.2
O2—S1—N1	104.21 (19)	C8—C13—C12	119.4 (3)
O1—S1—C8	109.00 (19)	C8—C13—H13A	120.3
O2—S1—C8	108.89 (18)	C12—C13—H13A	120.3
N1—S1—C8	105.39 (15)	C16A—C14—C15A	109.0 (13)
C2—N1—C7A	105.5 (3)	C16—C14—C17	109.4 (14)
C2—N1—S1	124.7 (3)	C16A—C14—C11	112.5 (8)
C7A—N1—S1	127.9 (3)	C16—C14—C11	115.5 (8)
C2—N3—C3A	104.6 (4)	C15A—C14—C11	109.1 (6)
N3—C2—N1	114.5 (4)	C17—C14—C11	110.1 (7)
N3—C2—H2B	122.7	C16—C14—C15	109.3 (12)
N1—C2—H2B	122.7	C17—C14—C15	104.1 (10)
C4—C3A—N3	128.7 (4)	C11—C14—C15	107.8 (7)
C4—C3A—C7A	119.9 (4)	C16A—C14—C17A	113.7 (12)
N3—C3A—C7A	111.4 (4)	C15A—C14—C17A	104.5 (10)
C5—C4—C3A	118.1 (4)	C11—C14—C17A	107.7 (7)
C5—C4—H4A	120.9	C14—C15—H15A	109.5
C3A—C4—H4A	120.9	C14—C15—H15B	109.5
C4—C5—C6	121.5 (4)	C14—C15—H15C	109.5
C4—C5—H5A	119.2	C14—C16—H16A	109.5
C6—C5—H5A	119.2	C14—C16—H16B	109.5
C7—C6—C5	121.4 (4)	C14—C16—H16C	109.5
C7—C6—H6A	119.3	C14—C17—H17A	109.5
C5—C6—H6A	119.3	C14—C17—H17B	109.5
C6—C7—C7A	116.8 (4)	C14—C17—H17C	109.5
C6—C7—H7A	121.6	C14—C15A—H15D	109.5
C7A—C7—H7A	121.6	C14—C15A—H15E	109.5
C7—C7A—C3A	122.3 (4)	H15D—C15A—H15E	109.5
C7—C7A—N1	133.7 (4)	C14—C15A—H15F	109.5
C3A—C7A—N1	104.0 (3)	H15D—C15A—H15F	109.5
C9—C8—C13	120.4 (3)	H15E—C15A—H15F	109.5
C9—C8—S1	120.0 (3)	C14—C16A—H16D	109.5
C13—C8—S1	119.7 (3)	C14—C16A—H16E	109.5
C8—C9—C10	119.6 (3)	H16D—C16A—H16E	109.5
C8—C9—H9A	120.2	C14—C16A—H16F	109.5
C10—C9—H9A	120.2	H16D—C16A—H16F	109.5
C9—C10—C11	122.0 (3)	H16E—C16A—H16F	109.5
C9—C10—H10A	119.0	C14—C17A—H17D	109.5
C11—C10—H10A	119.0	C14—C17A—H17E	109.5
C10—C11—C12	117.0 (3)	H17D—C17A—H17E	109.5
C10—C11—C14	121.3 (3)	C14—C17A—H17F	109.5
C12—C11—C14	121.7 (3)	H17D—C17A—H17F	109.5
C13—C12—C11	121.7 (3)	H17E—C17A—H17F	109.5
			
O1—S1—N1—C2	−158.3 (3)	O2—S1—C8—C9	22.1 (3)
O2—S1—N1—C2	−28.4 (4)	N1—S1—C8—C9	−89.2 (3)
C8—S1—N1—C2	86.2 (3)	O1—S1—C8—C13	−21.3 (3)
O1—S1—N1—C7A	39.6 (4)	O2—S1—C8—C13	−156.6 (3)
O2—S1—N1—C7A	169.5 (3)	N1—S1—C8—C13	92.1 (3)
C8—S1—N1—C7A	−75.9 (3)	C13—C8—C9—C10	0.2 (5)
C3A—N3—C2—N1	0.6 (5)	S1—C8—C9—C10	−178.5 (3)
C7A—N1—C2—N3	−1.4 (5)	C8—C9—C10—C11	−0.5 (5)
S1—N1—C2—N3	−166.9 (3)	C9—C10—C11—C12	0.5 (5)
C2—N3—C3A—C4	−178.8 (4)	C9—C10—C11—C14	179.2 (3)
C2—N3—C3A—C7A	0.5 (5)	C10—C11—C12—C13	−0.2 (5)
N3—C3A—C4—C5	−179.9 (4)	C14—C11—C12—C13	−178.8 (3)
C7A—C3A—C4—C5	0.8 (6)	C9—C8—C13—C12	0.1 (5)
C3A—C4—C5—C6	−1.2 (7)	S1—C8—C13—C12	178.8 (3)
C4—C5—C6—C7	0.3 (7)	C11—C12—C13—C8	−0.1 (5)
C5—C6—C7—C7A	0.9 (6)	C10—C11—C14—C16A	10.7 (19)
C6—C7—C7A—C3A	−1.2 (5)	C12—C11—C14—C16A	−170.7 (19)
C6—C7—C7A—N1	−178.1 (4)	C10—C11—C14—C16	174 (2)
C4—C3A—C7A—C7	0.4 (6)	C12—C11—C14—C16	−7(2)
N3—C3A—C7A—C7	−179.0 (3)	C10—C11—C14—C15A	131.8 (8)
C4—C3A—C7A—N1	178.0 (3)	C12—C11—C14—C15A	−49.7 (8)
N3—C3A—C7A—N1	−1.3 (4)	C10—C11—C14—C17	49.9 (10)
C2—N1—C7A—C7	178.8 (4)	C12—C11—C14—C17	−131.5 (10)
S1—N1—C7A—C7	−16.3 (6)	C10—C11—C14—C15	−63.0 (13)
C2—N1—C7A—C3A	1.6 (4)	C12—C11—C14—C15	115.6 (13)
S1—N1—C7A—C3A	166.4 (3)	C10—C11—C14—C17A	−115.3 (15)
O1—S1—C8—C9	157.3 (3)	C12—C11—C14—C17A	63.2 (15)

### Hydrogen-bond geometry (Å, °)

**Table:**

*D*—H···*A*	*D*—H	H···*A*	*D*···*A*	*D*—H···*A*
C7—H7A···O2^i^	0.93	2.56	3.430 (5)	156
C13—H13A···O1^i^	0.93	2.56	3.292 (5)	136

Symmetry codes: (i) −*x*+1/2, *y*+1/2, −*z*+1/2.
